# Pelvic actinomycosis presenting as a malignant pelvic mass: a case report

**DOI:** 10.1186/1752-1947-5-40

**Published:** 2011-01-27

**Authors:** Arife Simsek, Asiye Perek, Ibrahim Ethem Cakcak, Ali Vedat Durgun

**Affiliations:** 1 Istanbul University, Cerrahpasa School of Medicine, Department of General Surgery, 34098, Fatih, Istanbul, Turkey

## Abstract

**Abstract:**

**Introduction:**

Pelvic actinomycosis constitutes 3% of all human actinomycosis infections. It is usually insidious, and is often mistaken for other conditions such as diverticulitis, abscesses, inflammatory bowel disease and malignant tumors, presenting a diagnostic challenge pre-operatively; it is identified post-operatively in most cases. Here we present a case that presented as pelvic malignancy and was diagnosed as pelvic actinomycosis post-operatively.

**Case presentation**

A 48-year-old Caucasian Turkish woman presented to our clinic with a three-month history of abdominal pain, weight loss and difficulty in defecation. She had used an intra-uterine device for 16 years, however it had recently been removed. The rectosigmoidoscopy revealed narrowing of the lumen at 12 cm due to a mass lesion either in the wall or due to an extrinsic lesion that prevented the passage of the endoscope. On examination, there was no gynecological pathology. Magnetic resonance imaging showed a mass, measuring 5.5 × 4 cm attached to the rectum posterior to the uterus. The ureter on that side was dilated. Surgically there was a pelvic mass adhered to the rectum and uterine adnexes, measuring 10 × 12 cm. It originated from uterine adnexes, particularly ones from the left side and formed a conglomerated mass with the uterus and nearby organs; the left ureter was also dilated due to the pelvic mass. Because of concomitant tubal abscess formation and difficulty in dissection planes, total abdominal hysterectomy and bilateral salphingo-oophorectomy was performed (our patient was 48 years old and had completed her childbearing period). The cytology revealed inflammatory cells with aggregates of *Actinomyces*. Penicillin therapy was given for six months without any complication.

**Conclusions**

Pelvic actinomycosis should always be considered in patients with a pelvic mass especially in ones using intra-uterine devices, and who have a history of appendectomy, tonsillectomy or dental infection. Surgeons should be aware of this infection in order to avoid excessive surgical procedures.

## Introduction

Actinomycosis is a chronic granulomatous disease caused by any of several anaerobic organisms from the genus *Actinomyces*. Abdominal disease is usually insidious and is often mistaken for other conditions such as diverticulitis, abscesses, inflammatory bowel disease and malignant tumor, presenting a diagnostic challenge pre-operatively [1]; it is identified post-operatively in most cases [1]. Here, we present a case considered as pelvic malignancy and diagnosed as pelvic actinomycosis post-operatively.

### Case presentation

**Figure 1:**
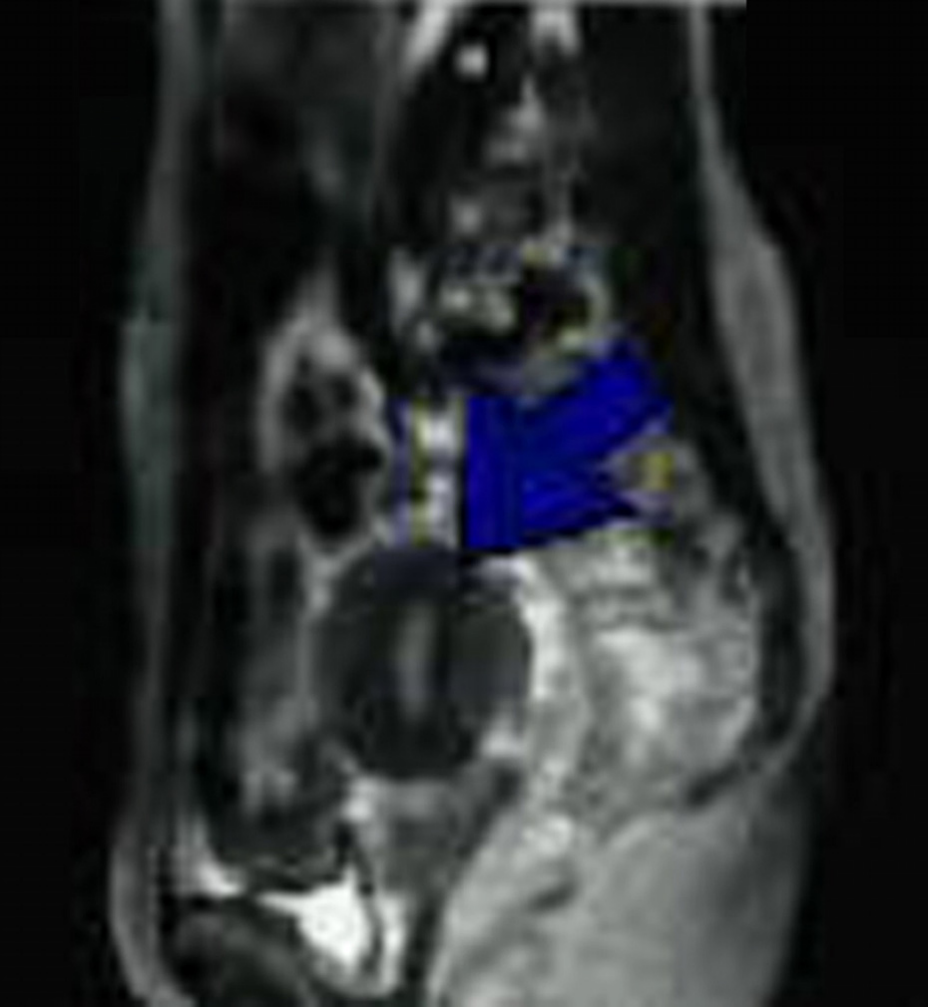
**Abdominal MRI shows a 5.5 × 4 cm mass near the rectum posterior to the uterus**.

A 48-year-old Caucasian Turkish woman presented to our clinic with a three-month history of abdominal pain, weight loss and difficulty in defecation. She had a 16-year history of intra-uterine device (IUD) use, which had recently been removed. On physical examination, the abdomen was normal, without any mass; on rectal digital examination, there was a rigid, immobile mass in the anterior part of the rectum. Laboratory evaluation showed a white blood cell count of 12,400. A rectosigmoidoscopy revealed narrowing of the lumen at 12 cm due to a mass lesion either in the wall or due to an extrinsic lesion that prevented the passage of the endoscope. Histopathological diagnosis of tissue samples taken from the mucosa of the narrowed lumen was inflammatory pattern. On gynecological examination and endovaginal ultrasound there was no gynecologic pathology. Magnetic resonance imaging (MRI) showed a mass, measuring 5.5 × 4 cm, attached to the rectum posterior to the uterus. The ureter on that side was dilated (Figure 1).

We performed an exploratory laparotomy, which showed a pelvic mass adhered to the rectum and uterine adnexes, measuring 10 × 12 cm. It originated from uterine adnexes particularly from the left side and formed a conglomerated mass with the uterus and nearby organs; the left ureter was also dilated due to the pelvic mass. Because of concomitant tubal abscess formation and difficulty in dissection planes, total abdominal hysterectomy and bilateral salphingo-oophorectomy was performed (our patient was 48-year-old and had completed her childbearing period).

Her post-operative recovery was normal. Cytology revealed inflammatory cells with aggregates of *Actinomyces*. Penicillin therapy was given for six months without any complication. She is well and has gained weight after one year.

## Discussion

Actinomycosis is a chronic granulomatous disease caused by any of several anaerobic organisms from the genus *Actinomyces*. Although previously thought to be a fungal infection, these organisms are Gram-positive, filamentous bacteria. Disease in humans is most commonly caused by *A. israelii *[2]. These organisms are not considered particularly virulent pathogens, but rather as opportunistic ones, because infection usually occurs only after disruption of the mucous membranes. The disease spreads by direct extension into surrounding tissues regardless of tissue planes through the formation of sinus tracts that can lead directly to the skin. Typically sulfur granules can drain from these tracts.

Actinomycosis is traditionally divided into three forms: cervicofacial, thoracic, and abdominogenital. The most frequent site of human infection is the cervicofacial area, accounting for about 40 to 50% of cases. Approximately, 15% of actinomycosis occurs in the thorax [2,3]. Twenty percent of actinomycotic infections occur in the abdomen and pelvis [3,4]. Pelvic actinomycosis constitutes 3% of all human actinomycotic infections.

Abdominal disease usually results from clinical or sub-clinical disruption of bowel mucosa. It often occurs as a firm mass that appears fixed to the surrounding tissue and can be mistaken for a tumor [4-6]. Abdominal surgery, ruptured viscus, tubo-ovarian abscess and IUDs are recognized risk factors for abdominal and pelvic actinomycosis [7]. *A. israelii *infects 1.65% to 11.6% of IUD users, and infection is more common in women who have had an IUD use in situ longer than four years. In females, *Actinomyces *is thought to be induced by oro-genital contact [8].

When pelvic actinomycosis occurs, it usually causes endometritis, salpingo-oophoritis, or tubo-ovarian abscess and a mass in the adnexa might be palpable, suggesting a pelvic malignancy [6,9,10]. Ultimately, extension to the abdominal wall or deep pelvic structures can occur. Primary bowel involvement is rare, although it has increased in frequency over recent years. The most common sites of the disease are the transverse colon and the cecum with the appendix [11,12].

Diagnosis of actinomycosis can be difficult because of the insidious nature of the infection. Usually, diagnosis is impossible pre-operatively, even after fine-needle aspiration [13]. The finding of sulfur granules from any other site than the tonsils is considered pathogonomonic [14]. Computed tomography- or ultrasound-guided biopsy can be used to obtain material for diagnosis. Occasionally, as in our patient, surgery may be required.

Penicillin is the drug of choice; resistance is rare. High doses must be given for prolonged courses. In those who are allergic to penicillin, options include tetracyclines, erythromycin, doxycycline and clindamycin. However, response to tetracyclines and ciprofloxacin is poor and a beta lactam antibiotic combined with a beta lactamase inhibitor, should be the first choice [15]. Parenteral therapy might be required for severe infection before changing to the oral route. Generally, the disease is treated until there is evidence of complete resolution. Surgery is occasionally required to drain abscesses, but because actinomycotic infection does not follow tissue planes, surgery can be complicated and, if possible, should be delayed at least until after a course of antibiotic use.

## Conclusions

Pelvic actinomycosis should always be considered in patients with pelvic mass especially in those using IUD, and who have a history of appendectomy, tonsillectomy or dental infection. Antimicrobial therapy should be initiated following surgery. Surgeons should be aware of this infection in order to avoid excessive surgical procedures.

## Abbreviations

*A. israelii*: *Actinomyces israelii*; CT: computed tomography; IUD: intra-uterine device, MRI: magnetic resonance imaging.

## Consent

The patient provided written consent for the publication of this case report and any accompanying images. A copy of the written consent is available for review by the journal's Editor-in-Chief.

## Competing interests

The authors declare that they have no competing interests.

## Authors' contributions

All authors planned to produce such a case report, analyzed and interpreted the patient data, reviewed the literature to design it and revised it for final approval of the version to be published.
